# Static sitting posture control during writing tasks in idiopathic scoliosis among freshmen

**DOI:** 10.1186/s13018-023-04228-z

**Published:** 2023-09-28

**Authors:** Qing Xia, Xinpeng Chen, Huanxia Wei, Guoliang Zhou, Jingmei Dong

**Affiliations:** 1 Department of Physical Education, Tongji University, Shanghai, 200092 China; 2 School of Engineering Sciences in Chemistry, Biotechnology and Health, KTH Royal Institute of Technology, 100 44 Stockholm, Sweden; 3 Shanghai YangZhi Rehabilitation Hospital (Shanghai Sunshine Rehabilitation Center), School of Medicine, Tongji University, Shanghai, 200092 China; 4 Innnovation Center PACE, Tongji University, Shanghai, 201804 China

**Keywords:** Adolescent idiopathic scoliosis, Posture control, Body pressure, Static sitting

## Abstract

**Background:**

The posture control deficit is one important dysfunction in adolescent idiopathic scoliosis (AIS) patients, which is related to the development of the disease. However, it is not apparent whether AIS could affect static sitting posture control in late adolescence.

**Objective:**

This study aims to compare static sitting posture control in idiopathic scoliosis freshmen with normal peers to reveal possible differences in posture stability between them during writing tasks.

**Methods:**

In total, there were 10 AIS patients and 11 normal college students chosen for the writing task test. Data on the distribution of gluteal pressure during sitting were gathered. The comparison between these two groups was made using the independent sample t-test.

**Results:**

The total excursion (TE) of the center of pressure (COP) of the AIS group considerably increased in comparison with the control group (CON) (*p = *0.029). The AIS group's average COP velocity in the anteroposterior (AP) direction was significantly higher than the CON group (*p = *0.048). The peak gluteal pressure on the right side was significantly higher in the AIS group than in the CON group (*p = *0.039). The right gluteal contact area dynamic variation was significantly higher in the AIS group compared to the CON group (*p = *0.025).

**Conclusions:**

AIS patients showed increased gluteal pressure and lower sitting posture stability during writing tasks.

## Introduction

Adolescent idiopathic scoliosis (AIS) is one kind of scoliosis that refers to patients over ten years of age with unclear pathogenesis [1]. Due to the imbalance of bilateral spinal muscle of AIS patients, more neuromuscular control was needed to complete the writing tasks during long periods of sitting posture [2]. AIS sufferers had to write in an awkward sitting posture because of their unequal shoulders, scapulae tilt, lumbar kyphosis, and pelvic tilt, all of which were brought on by spinal deformity [3]. Sitting for long periods in adolescence could trigger posture imbalances, increasing pressure between the disks and creating future risks of the spine [4], and may pose a great challenge to the sitting posture of the spine among AIS [5–7].

Previous research showed that teenagers with and without spinal deformities exhibited glaring discrepancies in their sitting posture control [8]. Center of pressure (COP) was commonly applied to assess posture control [9]. Changes in COP measurements indicated the approach the body develops to maintain posture stability in the context of the central posture control system that was supposed to be measured by COP variability [9]. Kim et al. [10] found that people with spinal deformities had a higher COP than the normal people, which indicated the people with spinal deformities had poorer posture control. Previous studies also showed that sitting control strategies in patients with scoliosis correlate with anteroposterior (AP) direction instability [11, 12].

Besides, the gluteal pressure distribution was applied to evaluate the control of sitting posture by several scholars [2]. Lee and Park [8] demonstrated that adolescents with spinal deformities had clear gluteal pressure asymmetry. Jung et al. [13] discovered that the pelvic tilt and the spinal deformity with C-sharped curves could result in an unbalanced sitting pressure. Patel et al. [14] suggested that severe thoracic scoliosis and pelvic tilt increased gluteal pressures on average, at their peak, and in broader areas. These studies indicated that AIS adolescents performed worse in posture control than normally developing adolescents.

Several previous studies focused on how AIS could affect standing posture control [15]. Few pieces of research focused on how AIS could affect sitting posture control, especially among late adolescent patients. Adolescent scoliosis develops between the ages of 11 and 18, and the patient's sitting posture control is still evolving [16]. First-year college students, who are called freshmen aged 18–19, are in their late adolescence according to the United Nations, and AIS-induced habitual sitting has entered a stable phase. It is still not apparent whether AIS could affect static sitting posture control during writing tasks in a stable phase [16]. This study aims to compare static sitting posture control in first-year AIS college students with normal peers to reveal possible differences in posture stability during writing tasks.

## Methods

### Subjects

In total, 502 college students participated in the spinal screening test at Tongji University in Shanghai, China. Twenty-one subjects were included in this experiment voluntarily. The measuring technique rigorously protected the privacy of the individuals, and only academic use was made of the data. All participants provided their written informed permission.

Basic physical data of participants were recorded, including their gender, age, height, weight, angle of trunk inclination (ATI), and AIS curve pattern. The Jiansheng Scoliometer (ATR-2, SanDoc health consulting Co., Ltd., Shanghai, China) was applied for the spine screening. In the spine screening, participants’ values of ATI ≥ 5° were considered to have a risk of scoliosis. SpineScan (Ad-Or Medical Technologies Ltd., Israel) was employed for spine evaluations. Participants were divided into two groups according to the ATI value. Ten participants’ values of ATI ≥ 7° with X-ray evidence of idiopathic scoliosis were included in the AIS group. Eleven participants’ values of ATI < 7° were included in the CON group. The inclusion criteria for subjects were 18 to 19 years of age studying at the university. Exclusion criteria were prior orthopedic surgery and spinal disease other than AIS.

Basic spine parameters were collected: ATI, body balance, and ROM. ATI was the main clinical index for detecting scoliosis [17]. It evaluated the asymmetry to indicate the degree of abnormal curvature of the spine (SSIFU002 version 01 SpineScan user manual). Body balance showed asymmetries in the heads, shoulders, and hips (SSIFU002 version 01 SpineScan user manual). ROM was a parameter used to gauge how well the spine could move (SSIFU002 version 01 SpineScan user manual). The participants’ basic data were shown in Table 1.

**Table 1:** Characteristics of the participants (mean ± SD)

Sex (male/female)	Control group (CON) (*n* = 10)	Study group (AIS) (*n* = 11)	*p*-value
	Male = 5 and female = 5	Male = 2 and female = 9	
Age (years)	18.30 ± 0.48	18.27 ± 0.46	0.90
Height (m)	1.68 ± 0.08	1.64 ± 0.07	0.21
Body mass (kg)	56.46 ± 7.16	52.57 ± 9.84	0.32
ATI	2.70 ± 0.82	7.82 ± 1.47	< 0.001
AIS curve pattern		36.4% L/R thoracic	
		63.6% L/R lumbar	

Values are expressed as mean ± standard deviation. The *p*-values are the results of between-group comparison of the respective data (t-test). Abbreviations: ATI, angle of trunk inclination; SD, standard deviation; AIS, adolescent idiopathic scoliosis

### Procedure

The sitting posture evaluation among participants during the writing tasks was done. The height of the study table was fixed at 67 cm, and the height of the study chair was fixed at 42.5 cm, which was fixed based on the 50th percentile among the subjects. Additionally, the study chair with a plastic surface did not have a sitting depth. Three hours before the experiment, participants were informed to be at rest and not have any writing obligations. The moment began when students began writing assignments while seated in the study chair in front of the study table. Without any previous notice, the data collection started and lasted for 40 s for each participant. The middle 20 s data were selected for accurate analysis. During the whole procedure, participants had to wear thin clothes.

### Data collection and analysis

**Fig. 1:**
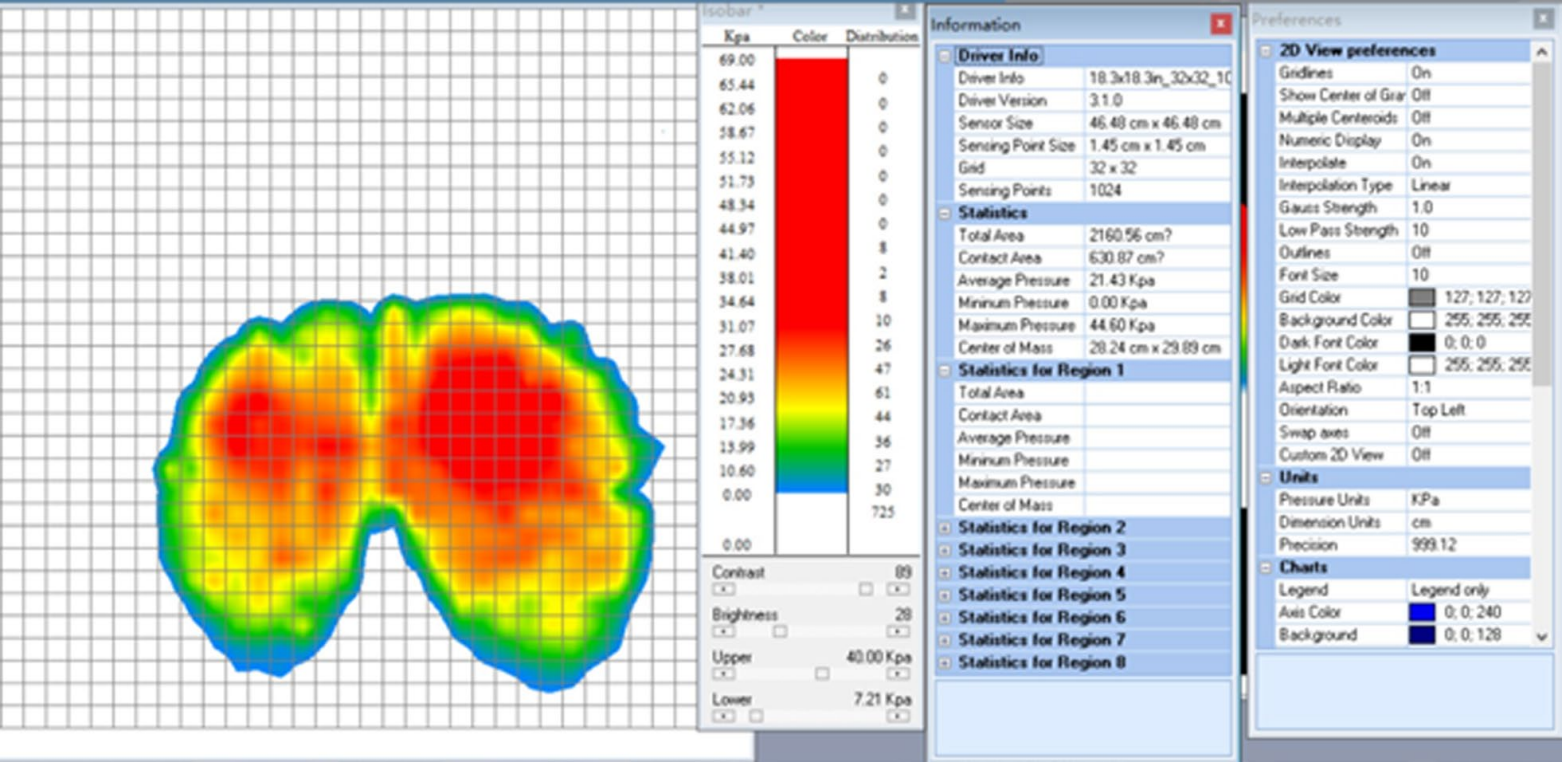
Gluteal pressure collection. It showed the gluteal pressure distribution of an AIS subject in this study

The Tactilus pressure sensor (Sensor Products Inc., Madison, New Jersey, USA) was used to measure the gluteal pressure under the sitting posture during writing tasks. The range of the pressure sensor was 0 to 34.664 kPa. The gluteal pressure distribution is shown in Fig. 1.

The MATLAB (MathWorks, Inc., Natick, USA, R2018a) scripts were used to measure the above raw data. The middle 20 s of the 40 s of data collected in the procedure were intercepted by MATLAB for further analysis. The pressure distribution was categorized into: COP, the peak gluteal pressure, and the gluteal contact area. The further processed key parameters and their significance are as follows:

*The total excursion (TE) of the COP* This parameter is the total distance covered by the COP throughout the trial. It stood for the posture control system's capacity to maintain balance [9].

*Average COP velocity in AP and ML directions* This parameter referred to the ratio of the COP route length to the trial time [18]. Increases in COP velocity were regarded to indicate a lower capacity to control posture [9].

*Peak gluteal pressure (left and right sides)* Peak gluteal pressure recorded the maximum pressure on the left and right hips, respectively. It was normalized by the weight of participants for accurate analysis [2].

*The difference value of the peak gluteal pressure between the right and left sides* This parameter measured the pressure imbalance between the left and right sides of the hips. It was normalized by the weight of participants for accurate analysis [2].

*Gluteal contact area (left and right sides)* The gluteal contact area measured the contact area between the left and right hips on the seat.

*Dynamic variance of the gluteal contact area (left, right side, and total)* This parameter measured the variation of the gluteal contact area during the trial.

### Statistical analysis

The Shapiro–Wilk test was used for assessing the normality for all variables [19]. All variables were from normal distribution. Then compared between groups, independent samples t-tests with Levene’s tests for homogeneity of variance were applied [20], with the statistical significance threshold set at 0.05. The data were processed using the SPSS 28.0 application for Windows (SPSS Inc., Chicago, USA).

## Results

Table 2 shows the body balance and ROM of the participants, which indicated their spine conditions.

**Table 2:** Body balance and the range of motion of the participants

Subject No	Body balance (°)			ROM (°)—lateral bending						ROM (°)—Flex/Ext						ROM (°)—rotation					
	Cervical	Thoracic	Lumbar	Cervical		Thoracic		Lumbar		Cervical		Thoracic		Lumbar		Cervical		Thoracic		Lumbar	
				Left	Right	Left	Right	Left	Right	Ant	Post	Ant	Post	Ant	Post	Left	Right	Left	Right	Left	Right
1	− 1	+ 2	− 1	45	43	24	28	16	23	45	23	61	30	43	15	80	80	23	26	21	25
2	0	+ 4	+ 1	41	24	22	23	22	21	45	28	44	30	45	10	80	80	18	29	24	27
3	+ 1	0	0	29	32	29	22	25	26	44	30	47	29	45	10	71	64	20	17	24	25
4	− 1	0	− 1	45	45	22	29	22	30	45	20	89	25	45	19	59	79	23	18	23	27
5	0	0	+ 1	39	27	22	22	24	30	44	38	76	13	46	14	77	75	23	26	28	21
6	− 1	− 2	− 1	40	45	27	21	28	30	45	16	69	30	43	19	80	80	28	27	17	15
7	− 1	+ 1	− 1	44	41	30	30	30	29	45	41	55	30	50	15	74	72	30	24	19	22
8	0	+ 1	− 1	45	45	26	27	30	24	44	39	86	30	50	16	80	80	24	18	15	18
9	− 2	0	− 3	42	45	30	30	13	20	45	41	77	30	48	14	80	74	22	24	17	21
10	+ 2	0	+ 3	40	45	30	30	19	22	45	58	83	25	50	20	80	74	23	23	22	28
11	0	− 1	0	39	42	30	23	26	25	43	57	68	25	50	18	77	79	24	30	26	24
12	0	0	0	41	36	16	13	11	12	42	21	68	28	40	17	80	79	29	28	12	12
13	0	0	− 1	44	43	18	20	12	16	45	35	90	15	39	18	73	80	26	20	16	14
14	0	− 1	− 2	45	44	30	30	22	23	45	42	77	23	49	17	69	80	18	28	24	29
15	− 1	− 2	0	43	38	24	24	20	17	45	43	90	13	49	13	68	64	30	24	28	27
16	0	0	− 1	41	38	10	15	29	30	45	27	49	30	37	16	80	69	29	27	24	17
17	+ 4	− 1	− 1	28	35	30	26	24	27	33	29	61	22	48	11	70	56	16	19	23	22
18	0	− 1	− 1	40	42	25	19	12	10	45	22	78	30	37	14	76	74	19	26	10	16
19	− 1	− 1	+ 1	43	31	16	26	23	15	43	22	75	20	40	12	61	55	21	18	18	19
20	− 3	− 2	− 1	39	45	20	19	26	29	45	36	47	27	50	18	68	72	23	26	21	21
21	− 1	+ 1	+ 1	43	45	26	29	20	26	45	14	90	15	43	12	80	78	24	30	23	21

+ Equals right, − Equals left. *Flex* flexion, *Ext* extension, *Ant* anterior, *Post* posterior

**Fig. 2:**
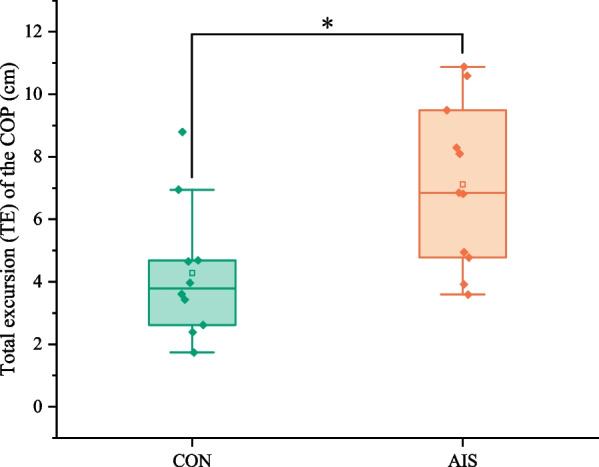
Comparison of the total excursion (TE) of the COP between the control group and the AIS group. CON, control group; AIS, adolescent idiopathic scoliosis group. *: Statistically significant, *p* < 0.05

The TE of COP differences between the AIS group and the CON group is presented in Fig. 2. When compared to the CON group, the TE of the COP of the AIS group significantly increased (*p = *0.029).

**Fig. 3:**
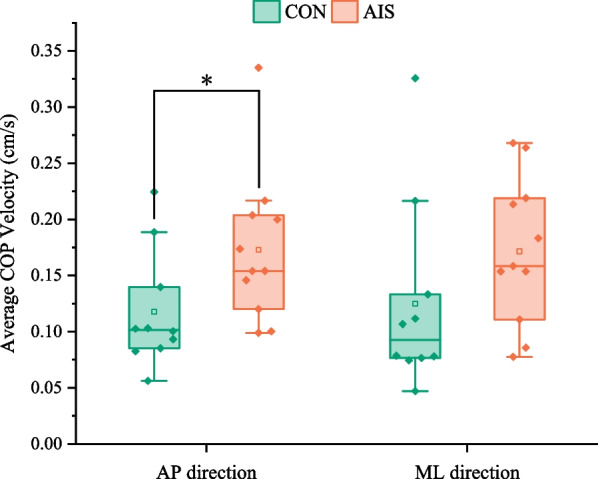
Comparison of the average COP velocity in different directions between the control group and the AIS group. CON, control group; AIS, adolescent idiopathic scoliosis group; AP, anterior–posterior; ML, medial and lateral. *Statistically significant, *p* < 0.05

The typical COP velocity in various directions is displayed in Fig. 3. In comparison with the CON group, the average COP velocity in the AP direction of the AIS group was significantly higher (*p = *0.048). In contrast, there was no difference between the AIS group and the CON group in terms of the average COP velocity in the ML direction (*p = *0.171).

**Fig. 4:**
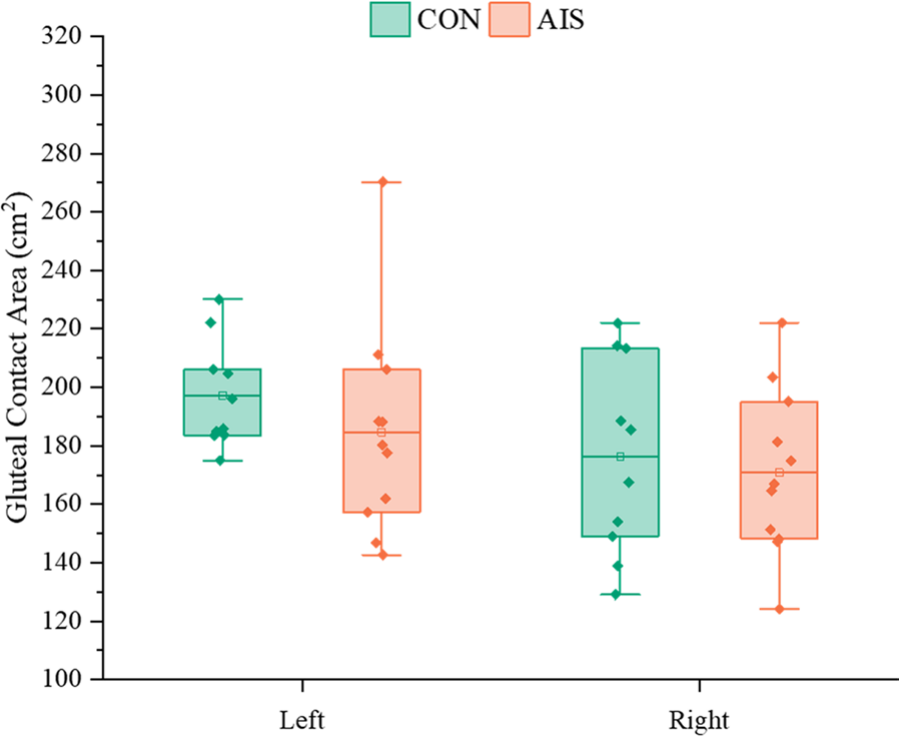
Comparison of the gluteal contact area on the left and right sides between the control group and the AIS group. CON, control group; AIS, adolescent idiopathic scoliosis group

Additionally, there was no difference in the left (*p = *0.333) and right (*p = *0.697) gluteal contact areas between the AIS group and CON group (Fig. 4).

**Fig. 5:**
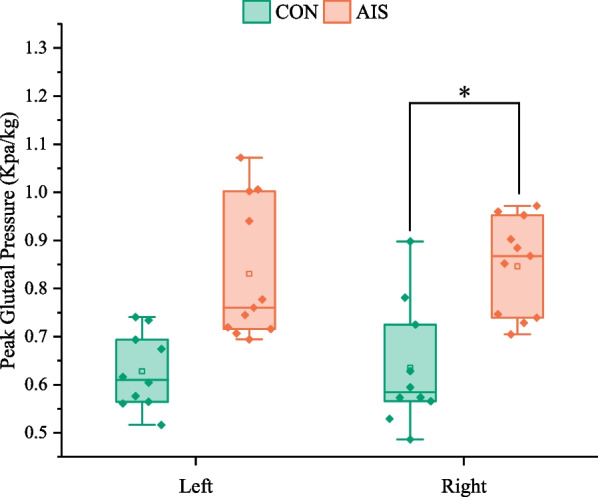
Comparison of the peak gluteal pressure on the left and right sides between the control group and the AIS group. CON, control group; AIS, adolescent idiopathic scoliosis group. *Statistically significant, *p* < 0.05

As demonstrated in Fig. 5, the right-side peak gluteal pressure was significantly higher in the AIS group than in the CON group (*p = *0.039). It is interesting to note that there was no difference between the AIS group and the CON group in the peak gluteal pressure on the left side (*p = *0.087).

**Fig. 6:**
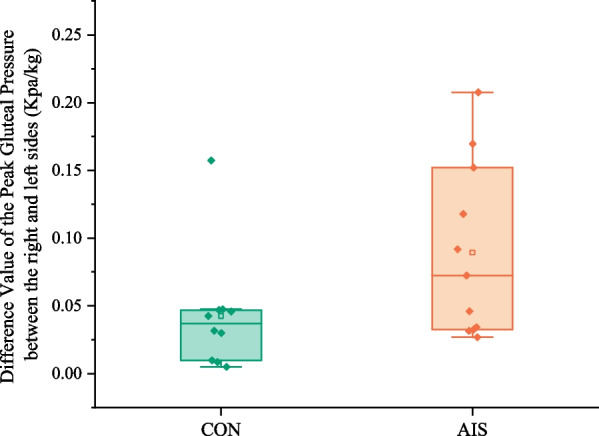
Comparison of the difference value of the peak gluteal pressure between the right and left sides between the control group and the AIS group. CON, control group; AIS, adolescent idiopathic scoliosis group

In Fig. 6, there was no difference between the right and left sides of the AIS group and CON group in terms of the peak gluteal pressure differential value (*p = *0.108).

**Fig. 7:**
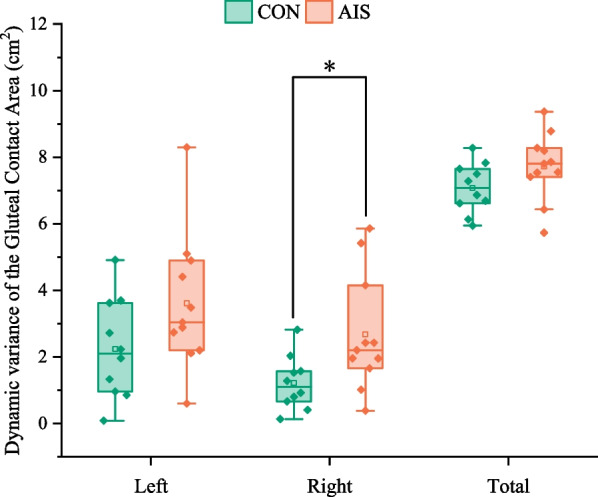
Comparison of the dynamic variance of the gluteal contact areas on the left side, on the right side, and in total between the control group and the AIS group. CON, control group; AIS, adolescent idiopathic scoliosis group. *Statistically significant, *p* < 0.05

Variations in the dynamic variance of the gluteal contact areas are shown in Fig. 7. In the AIS group compared to the CON group, the dynamic variation of the right gluteal contact area was significantly higher (*p = *0.025). Interestingly, there was no difference between the AIS group and CON group in the dynamic variation of the left (*p = *0.097) and total (*p = *0.117) gluteal contact area.

## Discussion

The purpose of this study is to compare sitting posture control in AIS students and their normal peers during static writing tasks. The results of some key parameters showed that AIS patients and normal students have differences in static sitting during writing tasks [8], which include: the TE of the COP, the peak gluteal pressure on the right side, the average velocity of COP in the AP direction, and the dynamic variance of the right gluteal contact area. Additionally, the results showed that posture control of AIS during writing tasks was not related to the following parameters: the gluteal contact area and the difference value of the peak gluteal pressure between the right and left sides.

The TE of COP was greater in AIS patients compared with normal college students, which was consistent with the previous research conclusion of Kim et al. [10]. This indicates that AIS adjusted their postures more frequently when they performed static writing tasks and had poor posture stability. The velocity of COP in the AP direction was significantly higher in the AIS group than in the CON group, which confirmed that scoliosis was related to the sitting stability in the AP direction [11, 12]. This indicates that AIS patients have worse posture control when completing writing tasks in a static sitting position compared with normal students in the sagittal plane. However, the velocity of COP in the ML direction did not differ significantly between the two groups. In the writing task, subjects relied performing more on the sagittal than the frontal plane, so this could be the possibility to explain why the velocity of COP in the AP direction differed significantly between the two groups but not in the ML direction. It is possible to determine whether AIS affects posture control in different motion planes by changing specific tasks.

The peak gluteal pressure was greater in AIS patients compared with normal college students, which was consistent with the previous research conclusion of Kim et al. [10]. This indicates that AIS put more pressure on their bodies when they performed static writing tasks. Additionally, there were significant differences in peak gluteal pressure on the right side, but no difference on the left side, which may attribute to the curve type of AIS or the specific writing task with the right hand. Thus, we can categorize scoliosis and further investigate whether pressure imbalance on both sides of the hips is related to the type of scoliosis. Furthermore, in this study, the difference value of the peak gluteal pressure between the right and left sides was smaller in normal college students than in AIS patients, which was consistent with the conclusions of previous studies by Jung, J.Y. et al., Lee and Park [8].

The dynamic variance of the right gluteal contact area in the AIS group was significantly higher than that in the normal group, indicating that the right hip moved more frequently in the AIS group than in the normal group during writing tasks. However, the dynamic variance of the left and the total gluteal contact area in the AIS group was not significantly different from that in the CON group, suggesting that the left hip movement and overall hip movement in the AIS group during the writing task were comparable to that in the normal group. Interestingly, the results of different dynamic variances of gluteal contact area parameters were not the same between the AIS group and the normal group, which could be related to a specific task. It can be considered to change the tasks under static sitting posture to further verify whether the dynamic variance of the gluteal contact area is a key parameter of the stability of AIS posture control.

The study did not consider the effect of scoliosis type on posture control. Additionally, only writing tasks were studied in this research, and no other tasks were included. Future studies could further analyze the type of scoliosis in patients with scoliosis and different tasks to determine whether they could affect sitting posture control during writing tasks.

## Conclusions

We evaluated the pressure distribution in AIS patients and normal subjects during a writing task in a static sitting posture. The variation of COP in AIS patients was higher than that in normal students, especially in the sagittal plane. Additionally, the peak gluteal pressure was significantly higher in AIS patients than the normal peer. There was no big difference in the gluteal contact area and the difference value of the peak gluteal pressure between the right and left sides between AIS students and normal students. Consequently, we concluded that AIS led to posture instability in static sitting during writing tasks, and caused higher gluteal pressure in the sitting posture compared to normal people.

This paper suggests that AIS patients demand more posture control while maintaining writing tasks than normal students. Future research could select different tasks or different curve types to investigate posture control in different tasks and its relationship with curve types and severity. Additionally, this study can provide physical therapists with a basis for posture training for AIS patients.

## Data Availability

The raw supporting data used in this article will be made available by the authors, without undue reservation.
